# Harnessing Azelaic Acid for Acute Myeloid Leukemia Treatment: A Novel Approach to Overcoming Chemoresistance and Improving Outcomes

**DOI:** 10.3390/ijms26094362

**Published:** 2025-05-03

**Authors:** Silvia Di Agostino, Anna Di Vito, Annamaria Aloisio, Giovanna Lucia Piazzetta, Nadia Lobello, Jessica Bria, Emanuela Chiarella

**Affiliations:** 1 Department of Health Sciences, University Magna Graecia of Catanzaro, 88100 Catanzaroo, Italy; sdiagostino@unicz.it; 2 Department of Clinical and Experimental Medicine, University Magna Graecia of Catanzaro, 88100 Catanzaro, Italy; divito@unicz.it (A.D.V.);; 3 Department of Medical and Surgical Sciences, University Magna Graecia of Catanzaro, 88100 Catanzaro, Italy

**Keywords:** azelaic acid (AZA), acute myeloid leukemia (AML), apoptosis, Notch signaling, reactive oxygen species (ROS)

## Abstract

Azelaic acid (AZA), an aliphatic dicarboxylic acid (HOOC-(CH2)_7_-COOH), is widely used in dermatology. It functions as an inhibitor of tyrosinase, mitochondrial respiratory chain enzymes, and DNA synthesis, while also scavenging free radicals and reducing reactive oxygen species (ROS) production by neutrophils. AZA has demonstrated anti-proliferative and cytotoxic effects on various cancer cells. However, its therapeutic potential in acute myeloid leukemia (AML) remains largely unexplored. AML is a complex hematologic malignancy characterized by the clonal transformation of hematopoietic precursor cells, involving chromosomal rearrangements and multiple gene mutations. The disease is associated with poor prognosis and high relapse rates, primarily due to its propensity to develop resistance to treatment. Recent studies indicate that AZA suppresses AML cell proliferation by inducing apoptosis and arresting the cell cycle at the G1 phase, with minimal cytotoxic effects on healthy cells. Additionally, AZA exerts antileukemic activity by modulating the ROS signaling pathway, enhancing the total antioxidant capacity in both AML cell lines and patient-derived cells. AZA also sensitizes AML cells to Ara-C chemotherapy. In vivo, AZA has been shown to reduce leukemic spleen infiltration and extend survival. As our understanding of AML biology progresses, the development of new molecularly targeted agents, in combination with traditional chemotherapy, offers the potential for improved treatment outcomes. This review aims to provide a comprehensive synthesis of preclinical evidence on the therapeutic potential of AZA in AML, consolidating current knowledge and identifying future directions for its clinical application.

## 1. Introduction

Acute myeloid leukemia (AML) is an aggressive hematologic malignancy characterized by the uncontrolled proliferation of undifferentiated myeloid cells in the bone marrow. Despite advances in chemotherapy, targeted therapies, and stem cell transplantation, AML remains associated with high relapse rates and poor overall survival, particularly in older patients and those with high-risk genetic mutations. The emergence of chemoresistance highlights the need for novel therapeutic strategies to improve patient outcomes [1,2,3].

Azelaic acid (AZA) is a naturally occurring dicarboxylic acid widely known for its dermatological applications due to its anti-inflammatory, antioxidant, and antiproliferative properties. Recent studies suggest that AZA exerts potent antileukemic effects by inducing apoptosis, modulating redox balance, and regulating key signaling pathways such as Notch and PI3K/Akt. Furthermore, AZA has demonstrated the ability to enhance the cytotoxic effects of conventional chemotherapy agents like cytarabine (Ara-C), suggesting its potential as an adjunctive treatment for AML [4,5,6,7].

This review provides a comprehensive analysis of AZA’s mechanisms of action in AML, discussing its effects on cellular proliferation, oxidative stress, and immune modulation. By synthesizing current preclinical findings, we aim to highlight AZA’s therapeutic potential and future directions for its clinical application in AML treatment.

## 2. Exploring the Structure and Molecular Mechanisms of Azelaic Acid

### 2.1. Structure, Pharmacological Benefits, and Dietary Sources

**Figure 1:**
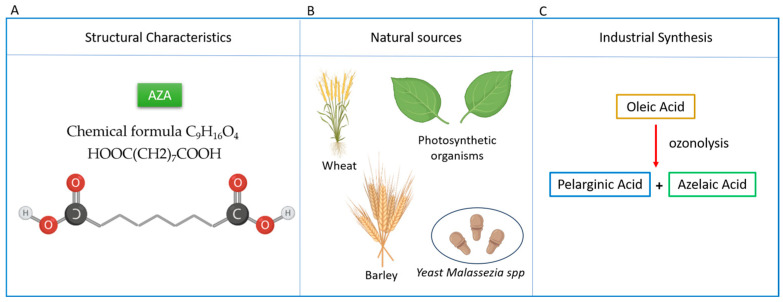
Overview of Azelaic Acid (AZA): Structure, Natural Sources, and Industrial Production. (**A**) AZA is classified as a dicarboxylic acid composed of 9 carbon atoms, 16 hydrogen atoms, and 4 oxygen atoms. The chemical formula C_9_H_16_O_4_ or HOOC(CH2)_7_COOH and, its IUPAC name is Nonanedioic acid. (**B**) Main natural sources of AZA include wheat, rye, and barley. It is also produced by plants and the skin commensal yeast *Malassezia* spp. (**C**) Industrially, azelaic acid is synthesized via ozonolysis of oleic acid, yielding both azelaic acid and pelargonic acid (nonanoic acid).

Azelaic acid (or azelate) (AZA) is a 1,7-heptanedicarboxylic acid with nine carbon atoms, known for its anti-inflammatory, antibacterial, antioxidant, and anti-keratinizing effects (Figure 1). These properties contribute to its various pharmacological applications in dermatology, making it effective in treating conditions such as papulopustular rosacea and acne vulgaris, among others [8].

Additionally, several studies have demonstrated that azelaic acid is nontoxic, nonteratogenic, and non-mutagenic in both in vitro and in vivo investigations [9]. For instance, in a study by Breathnach et al., azelaic acid showed no mutagenic potential in Ames tests or chromosomal aberration assays using mammalian cells. Furthermore, long-term in vivo studies demonstrated no teratogenic effects even at high doses, with no observed developmental abnormalities or maternal toxicity. These findings support its favorable safety profile, which has led to its widespread use in dermatology for chronic conditions such as rosacea and acne, often involving long-term application with minimal side effects [8,9,10,11].

From a biochemical perspective, it is classified as a medium-chain saturated dicarboxylic acid (DA) and is uniquely water-soluble due to the presence of two terminal carboxyl groups in its molecule. Unlike long- and medium-chain triglycerides, which are administered as emulsions, AZA has the advantage of being delivered via a peripheral vein in the form of inorganic salts [10,11] (Figure 1).

AZA naturally occurs in wheat, rye, and barley [8]. It is a common but insufficiently studied metabolite produced by photosynthetic organisms like plants [12,13] and phytoplankton [14]. Additionally, it is produced by the yeast *Malassezia* spp. (formerly *Pityrosporum ovale*), a skin commensal yeast responsible for the skin condition known as *Pityriasis (Tinea) versicolor* [15]. Currently, ozonolysis is the exclusive industrial process for sustainably producing azelaic acid, primarily using oleic acid [16].

### 2.2. Physiological Functions and Metabolism

In humans, AZA is synthesized through the omega-oxidation of fatty acids and is found in small amounts as a physiological component in urine. However, AZA is present in excess in the urine of patients with ketosis and those with congenital or acquired inability to β-oxidize monocarboxylic acids, a condition known as dicarboxylic acidosis [12,17] Being a dicarboxylic acid, AZA is produced through ω-oxidation of monocarboxylic acids when the β-oxidation of free fatty acids is compromised. Specifically, dicarboxylic acids (DAs) undergo β-oxidation in both peroxisomes and mitochondria via a carnitine-independent pathway. The β-oxidation of odd-chain DAs results in acetyl-CoA and malonyl-CoA, which cannot be further oxidized and are instead utilized in lipogenesis. Conversely, the β-oxidation of even-chain DAs produces acetyl-CoA and succinyl-CoA, with the latter serving as a precursor for gluconeogenesis [12,13]. However, the energy generated from the cellular oxidation of DAs is greater than that from glucose but less than that from long- or medium-chain fatty acids. For instance, the ATP/CO_2_ ratio of azelaic acid (with nine carbon atoms) is similar to that of palmitic acid (a 16-carbon saturated fatty acid) at 8.2 compared to 8.1, and notably higher than that of glucose at 6.3 [12,13]. AZA is attributed to multiple mechanisms of action resulting in antimicrobial, anti-inflammatory, antioxidant, and keratinolytic effects, including potential anticancer properties [18,19]. It exerts these effects through various mechanisms: (1) inhibiting enzymes like tyrosinase and, to a greater extent, thioredoxin; (2) negatively modulating several other oxidoreductive enzymes involved in DNA synthesis, such as DNA polymerase and mitochondrial respiratory chain oxidoreductases; (3) inhibiting microsomal 5α-reductase and suppressing anaerobic glycolysis; (4) acting as a scavenger of toxic oxygen species in vitro, particularly by inhibiting the production of reactive oxygen species by neutrophils [12,14,17].

### 2.3. Effects on Tyrosinase and Melanogenesis

Interestingly, AZA has been found to competitively suppress the +action of the tyrosinase enzyme in cultured human keratinocytes, melanocytes, melanoma cells, murine melanoma cells, and in purified enzymes from *Escherichia coli*, rat liver, and human melanoma [15,16]. The thioredoxin (TRX) system plays a critical role as a major cellular antioxidant pathway regulating redox balance. This system includes NADPH, thioredoxin reductase (TRXR), thioredoxin (TXN), and the negative regulator thioredoxin-interacting protein (TXNIP), also known as vitamin D3 upregulated protein 1, encoded by TXNIP. TXNIP binds to reduced TXN through intermolecular disulfide interactions, inhibiting its activity. TRXR, a selenoenzyme, uniquely utilizes reducing equivalents from NADPH generated by the pentose phosphate pathway (PPP) to maintain TXN in its reduced form. NADPH keeps thioredoxin reductase (TRXR) in its reduced state, facilitating the transfer of reducing equivalents to TXN. Reduced TXN then donates electrons to various cellular proteins.

**Figure 2:**
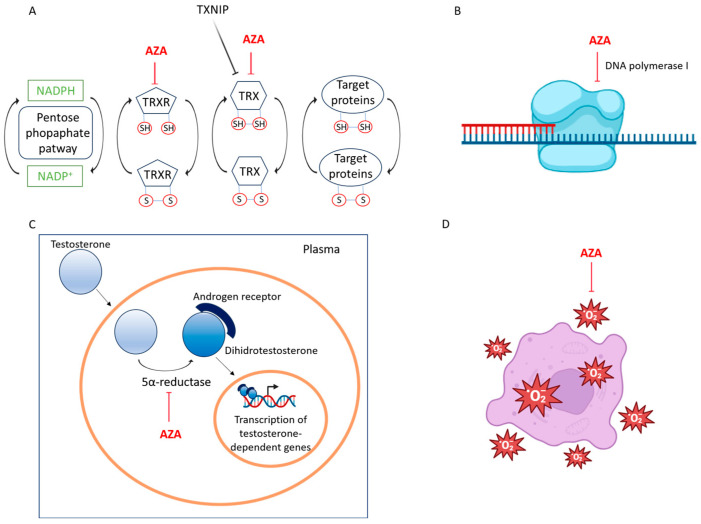
The molecular mechanisms underlying the activity of azelaic acid. (**A**) Azelaic acid is a reversible competitive inhibitor of thioredoxin (Trx) and thioredoxin reductase (TrxR). These enzymes are involved in reducing oxidized cysteine thiols in various proteins, thereby regulating cellular redox balance, survival, proliferation, DNA synthesis, transcription factor activity, and apoptosis. (**B**) Azelaic acid exhibits binding affinity and nonbonding interactions with the DNA polymerase I receptor (2KFN), resulting in the inhibition of its activity. (**C**) Azelaic acid acts as a competitive inhibitor of mitochondrial oxidoreductases and 5 alpha-reductase, thereby preventing the conversion of testosterone to 5-dehydrotestosterone and reducing inflammatory effects. (**D**) Azelaic acid mitigates inflammation by inhibiting the formation of free radicals produced by neutrophils and by reducing the effects of reactive oxygen species.

Through electron donation (e^−^), thioredoxin (TXN) supports several cellular functions: it aids peroxiredoxins (PRX) in scavenging hydrogen peroxide (H_2_O_2_), assists ribonucleotide reductase (RNR) in generating 2′-deoxyribonucleotides (dNTPs) for DNA [20]. AZA, by binding TRXR and TXN, functions as a skin-whitening agent by inhibiting tyrosinase activity and by disrupting DNA synthesis and mitochondrial activity in hyperactive and abnormal melanocytes [16,18] (Figure 2A).

Furthermore, AZA directly inhibits tyrosinase activity, which is a key enzyme for melanogenesis [19]. This enzyme plays a crucial role in converting tyrosine into DOPA and DOPA-quinone, which are precursors to melanin [12,21] In tissue culture, AZA induces a cytotoxic effect on malignant melanocytes, which depends on both the dosage and the duration of exposure. This effect is characterized by mitochondrial damage and the inhibition of DNA synthesis. Tumor cell lines lacking tyrosinase exhibit similar susceptibility. In contrast, normal cells exposed to equivalent concentrations of AZA, which are toxic to tumor cells, generally remain undamaged [20,22].

### 2.4. Inhibition of DNA Synthesis and Mitochondrial Function

Azelaic acid has also been shown to directly influence the activity of DNA polymerase I. Binding affinity and interaction studies have demonstrated that compounds such as 9-octadecenoic acid, along with drugs containing trifluoroacetyl and trifluoromethyl groups, are the most effective inhibitors of 2KFN (DNA polymerase I).

The observation that azelaic acid (AZA) directly influences DNA polymerase I activity is particularly significant in the context of drug development for proliferative diseases such as acute myeloid leukemia (AML) (Figure 2B). DNA polymerase I plays a key role in DNA replication and repair, processes that are often dysregulated in cancer cells. Inhibiting this enzyme can halt the rapid proliferation of malignant cells and increase their susceptibility to DNA-damaging agents. Molecular docking studies have shown that compounds with structural similarity to azelaic acid, such as 9-octadecenoic acid and those bearing trifluoroacetyl and trifluoromethyl groups, exhibit strong binding affinity to the enzyme’s active site. These findings provide a valuable scaffold for designing more potent DNA polymerase I inhibitors. By mimicking or enhancing the binding characteristics of AZA, it may be possible to develop novel therapeutic agents that selectively inhibit DNA replication in leukemic cells, while minimizing off-target effects in normal tissues. This mechanism complements AZA’s known actions on redox balance and cell cycle regulation, further underscoring its potential as a multi-targeted agent in cancer therapy [4,6,20].

### 2.5. Targeting Steroid Metabolism and Energy Production via 5α-Reductase and Glycolytic Pathway Suppression

Azelaic acid exerts its anti-infective and anti-inflammatory effects by competitively inhibiting mitochondrial oxidoreductases and 5α-reductase [12]. The steroid 5α-reductase (EC 1.3.99.5) enzyme catalyzes the NADPH-dependent reduction of the double bond in various 3-oxo-Δ^4^ steroids, facilitating the conversion of testosterone to 5α-dihydrotestosterone. In humans, this enzymatic activity is essential for male sexual differentiation and may contribute to conditions such as benign prostatic hyperplasia, alopecia, hirsutism, and prostate cancer [23]. By targeting 5α-reductase, azelaic acid exerts anti-androgenic and anti-seborrheic effects. Additionally, it demonstrates bacteriostatic activity against both aerobic and anaerobic bacteria [12,24] (Figure 2C).

### 2.6. Regulation of ROS Signaling and Antioxidant Defense

Azelaic acid scavenges reactive oxygen species (ROS) and other free radicals. Specifically, it inhibits ROS generated by neutrophils, thereby reducing oxidative tissue damage at sites of inflammation and suppressing melanin formation [12,25]. AZA further enhances this effect by downregulating cathelicidin activation through the inhibition of kallikrein-5 [26] (Figure 2D).

By inhibiting the activity of thioredoxin reductase (TrxR), reducing ROS generation, and hindering DNA synthesis, azelaic acid (AZA) has demonstrated antitumor effects on various cancer types, including lentigo maligna, malignant melanoma, lymphoma, and human T lymphotropic virus 1 (HTLV-1)-infected T-cell leukemia [5].

### 2.7. Anti-Inflammatory and Immunomodulatory Effects

It was reported that azelaic acid can reverse UV-induced inflammatory reactions in the skin, which may explain its effectiveness in treating conditions like rosacea, particularly in individuals whose symptoms are triggered by sunlight. In particular, AZA significantly reduced ultraviolet B (UVB)-induced nuclear translocation of the nuclear factor kB (NF-κB) p65 subunit and the phosphorylation of the p38 mitogen-activated and stress-activated protein kinase. Additionally, AZA activated peroxisome proliferator-activated receptor γ (PPARγ), which plays a crucial role in regulating inflammation [27,28].

In light of the above, Azelaic acid is widely used for several reasons: (1) it is significantly more affordable than other dicarboxylic acids; (2) it does not exhibit any obvious toxic, teratogenic, or mutagenic effects; (3) when administered orally to humans at the same concentrations as other dicarboxylic acids, it achieves much higher serum and urinary concentrations. The serum levels and urinary excretion resulting from intravenous or intra-arterial infusions of AA are considerably higher than those attainable through oral administration [9,10,29]. Considering the numerous benefits of azelaic acid, this review summarizes the limited evidence on its effects against hematopoietic cancer cells and explores its potential future applications in leukemia treatment.

## 3. Advancements and Challenges in Acute Myeloid Leukemia: From Genetic Insights to Therapeutic Strategies

**Figure 3:**
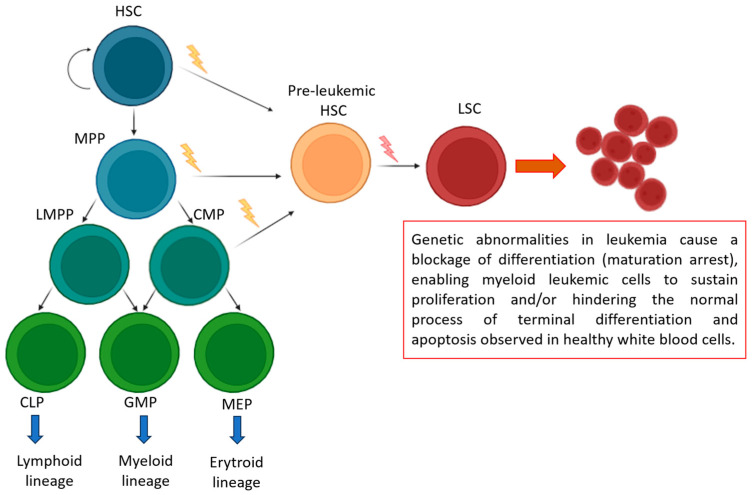
Genetic Lesions in Leukemia: Mechanisms of Differentiation Blockage and Cell Survival. During normal hematopoiesis (green box), quiescent hematopoietic stem cells (HSCs) with self-renewal capabilities generate multipotent progenitors (MPPs). These MPPs can then differentiate into lymphoid-primed multipotent progenitors (LMPPs), common myeloid progenitors (CMPs), common lymphoid progenitors (CLPs), granulocyte-macrophage progenitors (GMPs), and megakaryocyte-erythroid progenitors (MEPs). A restricted progenitor can be converted into a leukemic stem cell (LSC) through two consecutive mutations that grant self-renewal abilities.

Acute myeloid leukemia (AML) is a heterogeneous and often fatal bone marrow stem cell cancer characterized by the uncontrolled proliferation of hematopoietic stem cells (HSCs) or early committed myeloid progenitors [29]. This abnormal expansion of hematopoietic progenitor cells results in a blockage of myeloid differentiation, though the molecular mechanisms underlying this arrest remain largely unclear [30,31]. Mutations in hematopoietic stem cells (HSCs) and progenitor cells give rise to pre-leukemic stem cells, which can eventually transform into leukemic stem cells (LSCs). This transformation often occurs without a clinically identifiable pre-leukemic phase, though the development of acute leukemia typically requires multiple mutations. Additionally, a restricted progenitor cell can become an LSC through secondary mutations that confer self-renewal capabilities [32] (Figure 3).

Emerging research suggests that metabolic perturbations, driven by specific metabolites and their associated pathways, play a crucial role in leukemogenesis and disease progression. These alterations interact with underlying genetic, epigenetic, and functional factors, contributing to proleukemic processes—from cellular transformation to chemotherapy resistance [33,34] AML is the most common form of acute leukemia in adults, with a median diagnosis age of 68 years. It is often accompanied by complications such as infections, anemia, and bleeding [35,36].

The development of AML is influenced by various factors that contribute to leukemogenesis. External risk factors include high-dose radiation, chronic exposure to benzene, long-term tobacco smoking, and certain chemotherapeutic agents, such as alkylating agents and topoisomerase II inhibitors. These agents primarily induce DNA damage through oxidative mechanisms. Additionally, obesity is an internal risk factor that increases the likelihood of AML developing [37].

AML can also arise from the progression of other clonal disorders in hematopoietic stem cells, driven by genomic instability and the accumulation of additional mutations. Examples include myeloproliferative neoplasms (MPNs), which are marked by excessive blood cell production, and myelodysplastic syndromes (MDS), characterized by defective maturation and ineffective hematopoiesis AML can also arise from the progression of other clonal disorders in hematopoietic stem cells, driven by genomic instability and the accumulation of additional mutations. Examples include myeloproliferative neoplasms (MPNs), which are marked by excessive blood cell production, and myelodysplastic syndromes (MDS), characterized by defective maturation and ineffective hematopoiesis [38].

AML exhibits genetic diversity, characterized by various chromosomal abnormalities, mutations, gene expression patterns, and epigenetic profiles that define disease subtypes and distinct clonal populations within individual patients [1].

Historically, cytogenetic markers have been used to classify patients into three risk categories: favorable, intermediate, and unfavorable. These categories are now defined according to the latest frameworks provided by the 5th World Health Organization classification (WHO-2022), the 2022 European LeukemiaNet recommendations (ELN-2022), and the International Consensus Classification (ICC) [1,39,40,41].

Favorable-risk leukemias include core-binding factor leukemias with specific genetic abnormalities (t(8;21) and inv(16) or t(16;16)), as well as acute promyelocytic leukemia (t [15;17]). Additionally, acute myeloid leukemia with *NPM1* mutations (without *FLT3–ITD*) or in-frame *CEBPA* mutations in the basic leucine zipper region is now categorized as favorable-risk due to its heightened sensitivity to chemotherapy [1,40].

Intermediate-risk AML accounts for about 40% of cases, often involving patients with normal cytogenetics. The updated European LeukemiaNet 2022 guidelines now classify all AML cases with an *FLT3–ITD* mutation as intermediate risk, regardless of the *FLT3–ITD* allelic ratio or *NPM1* mutation status. This update reflects improved outcomes observed with FLT3 inhibitors in *FLT3–ITD*-positive, *NPM1* wild-type patients, emphasizing the importance of *NPM1* minimal residual disease (MRD) as a key prognostic factor in *FLT3–ITD* and NPM1 co-mutated cases [2].

The European LeukemiaNet 2022 guidelines also classify additional conditions as adverse-risk disease in acute myeloid leukemia (AML), including mutations in myelodysplastic syndrome-related genes (*ASXL1*, *RUNX1*, *BCOR*, *EZH2*, *SF3B1*, *SRSF2*, *STAG2*, *U2AF1*, and *ZRSR2*), and the cytogenetic fusion t(8;16) (*KAT6A*::*CREBBP*). These mutations can also appear in de novo AML cases and are associated with a poor prognosis [36].

In the past decade, there has been steady progress in drug development for AML, with a growing focus on genomic-based therapies. Induction chemotherapy, followed by consolidation with additional chemotherapy and/or an allogeneic stem cell transplant, has long been the primary treatment for AML. However, relapses are common, and the disease is ultimately fatal for most patients. Recent advancements, such as mutant isocitrate dehydrogenase (IDH) inhibitors, FLT3 inhibitors, and B-cell lymphoma 2 (BCL-2) inhibitors combined with hypomethylating agents (HMAs), have focused on treating relapsed/refractory disease or elderly patients. Despite these advancements, the outcomes of current therapies remain suboptimal, underscoring the need for novel treatment strategies. In this context, exploring natural compounds and bisphosphonates offers promising avenues for enhancing therapeutic efficacy and overcoming resistance in AML treatment [34].

In summary, azelaic acid demonstrates a broad spectrum of biological activities, including antioxidant, anti-inflammatory, and antitumor effects, through its interactions with multiple enzymatic targets and redox regulatory pathways. These mechanistic insights provide a solid biochemical foundation for its potential therapeutic application in oncology, particularly in AML.

## 4. Antileukemic Potential of AZA in Acute Myeloid Leukemia: Cellular Mechanisms and Immunomodulatory Effects

### 4.1. In Vitro Therapeutic Effects of Azelaic Acid on Acute Myeloid Leukemia Cells

AZA has demonstrated antitumor effects on certain cancer cells, including human cutaneous malignant melanoma and human choroidal melanoma [4,13,42]. However, with only three studies investigating its effectiveness on AML cells, scientific knowledge in this area remains quite limited.

In 2017, Pan et al. were the first to report the antileukemic activity of AZA in a panel of AML cell lines, including the well-differentiated U937, THP-1, and KG-1 cells, as well as the promyelocytic HL-60 and NB4 cells. AZA affected cell viability in a time- and dose-dependent manner, with the best results observed after three days of treatment. The IC50 values were 1.4 µM for U937, 1.2 µM for THP-1, 1.7 µM for KG-1, 1.3 µM for NB4, and 1.9 µM for HL-60 [4].

The following year, Dongdong and colleagues confirmed the reduced proliferation of U937, HL-60, and Molm-13 cells after exposure to AZA at 5.0 µM for 48 h. More interestingly, they extended these studies to primary cells from AML patients. In these cases, cell viability remained at approximately 44% for AML-M1, 12% for AML-M3, and 65% for AML-M5 patient-derived cells, respectively. These data suggest that AZA is particularly active in acute myeloid leukemia cells with intermediate differentiation grades. Furthermore, a cell cycle block at the G1/G0 phase was observed in HL-60 and U937 cells treated with AZA at 5 µM [6]. Additionally, AZA at a concentration of 3 µM significantly inhibited colony formation, reducing it by over 60% in NB4, HL-60, KG-1, U937, and THP-1 cells, highlighting its potent role in suppressing the clonal expansion of AML cells (Table 1) [4].

The functional efficacy of AZA in targeting myeloid leukemia cells appears to be highly specific. Healthy PBMCs exposed to high concentrations of AZA (5–10 µM) did not exhibit any cytolytic effects, while apoptosis rates ranged from 30% to 50% in all tested AML cell lines and AML patient cells dongdon [4,6]. AZA-induced apoptosis was evident through the loss of mitochondrial membrane potential (MMP) and phosphatidylserine externalization in HL-60 and U937 cells treated with 5.0 µM AZA for 24 h. The antileukemic activity of AZA against AML is mediated through modulation of the ROS signaling pathway. Specifically, AML cell lines (U937, HL-60, and THP-1) and AML patient cells treated with 5.0 µM AZA for 24 h exhibited reduced intracellular ROS levels, alongside an increase in total antioxidant capacity, including enhanced SOD and GSH activity (Table 1) [6].

AZA exerts its antileukemic effects not only directly on cancer cells but also indirectly by promoting the proliferation of immune effector cells. Treatment of NK and T cells with an optimal concentration of 10 µM AZA significantly increased their proliferation and the secretion of cytolysis-associated cytokines, including IFN-γ and TNF-α. The potent antileukemic effect of AZA-treated supernatants was validated in co-culture experiments with THP-1, U937, and human AML cells, showing a marked reduction in cell viability. Furthermore, AZA treatment enhanced the expression levels of cytolysis-related receptors in T cells (CD25 and CD69) and NK cells (TRAIL and CD107a), leading to their activation. Notably, the enhanced functional activity of AZA-treated NK cells in killing AML cells more efficiently than control cells was also observed at the single-cell level in a microfluidic chip, underscoring AZA’s ability to sensitize NK cells. These findings, as reported by Dongdong and colleagues, emphasize the crucial role of AZA in activating immune effector cells and stimulating the release of cytolysis-associated cytokines against AML cells (Table 1) [6]. In vitro studies have consistently shown that AZA effectively inhibits proliferation, induces apoptosis, and enhances antioxidant defense in AML cells, while sparing healthy cells. These findings support AZA’s role as a selective and potent antileukemic agent with additional immune-enhancing capabilities.

AZA exerts its antileukemic effects not only directly on cancer cells but also indirectly by promoting the proliferation of immune effector cells. Treatment of NK and T cells with an optimal concentration of 10 µM AZA significantly increased their proliferation and the secretion of cytolysis-associated cytokines, including IFN-γ and TNF-α. The potent antileukemic effect of AZA-treated supernatants was validated in co-culture experiments with THP-1, U937, and human AML cells, showing a marked reduction in cell viability. Furthermore, AZA treatment enhanced the expression levels of cytolysis-related receptors in T cells (CD25 and CD69) and NK cells (TRAIL and CD107a), leading to their activation. Notably, the enhanced functional activity of AZA-treated NK cells in killing AML cells more efficiently than control cells was also observed at the single-cell level in a microfluidic chip, underscoring AZA’s ability to sensitize NK cells. These findings, as reported by Dongdong and colleagues, emphasize the crucial role of AZA in activating immune effector cells and stimulating the release of cytolysis-associated cytokines against AML cells (Table 1) [6].

### 4.2. Mechanisms of AZA-Mediated Cytotoxicity in AML: Modulation of Notch Signaling, PI3K/Akt Pathway, and Redox Balance

**Figure 4:**
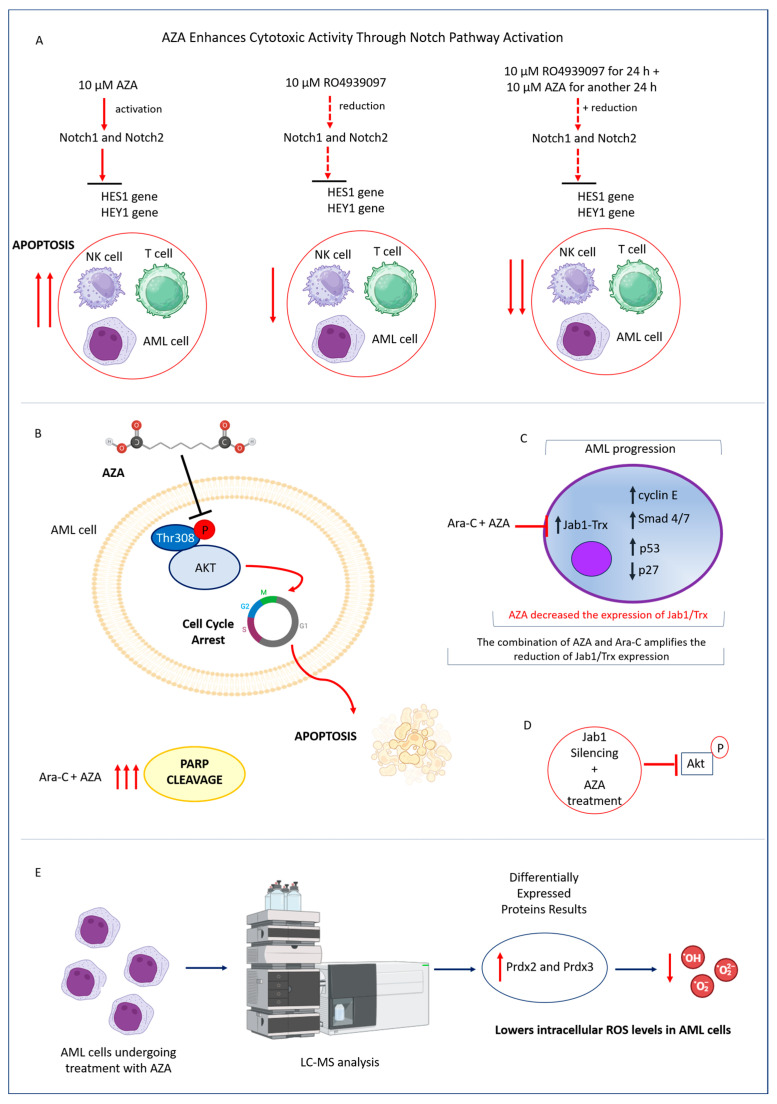
Effects of AZA on Notch Signaling, Jab1 Expression, and Redox Balance in AML Cells. (**A**) AZA treatment upregulated Notch1 and Notch2, activating the Notch signaling pathway. When AZA was combined with the Notch inhibitor RO4929097, the expression levels of Notch1, Notch2, and their downstream targets HES1 and HEY1 were reduced. This combination decreased AZA-induced apoptosis and mitigated its cytotoxic effects in AML cells. (**B**) AZA inactivated Akt at the Thr308 residue and triggered apoptotic poly (ADP-ribose) polymerase (PARP) activation in AML cells. This effect was further enhanced by the combined treatment with AZA and Ara-C. (**C**) In AML cells, AZA reduced the expression of Jab1, a key signaling molecule involved in tumorigenesis that regulates substrates including p27, cyclin E, Smad 4/7, and p53. This modulation primarily affects the Trx axis and is further amplified by the combined treatment with AZA and Ara-C. (**D**) Inhibition of Jab1 improved the effectiveness of AZA against AML cells, suggesting an interplay between Jab1, Akt, and Trx. (**E**) LC-MS analysis showed that AZA increased the expression of Prdx2 and Prdx3 in AML cells. The Prdx system was crucial for reducing intracellular ROS levels and maintaining redox balance.

AZA mediates cytotoxic activity on AML cells by modulating Notch signaling, an important regulator of immune system development and function [7,43]. Specifically, Notch1 and Notch2 proteins were found to be significantly upregulated after treatment with 10 µM AZA in both immune effector cells (NK and T cells) and in acute myeloid leukemia cell lines (THP-1, Molm-13), as indicated by Protein Mass Spectrometry Analysis and WEGO Analysis. Pharmacological inactivation of endogenous Notch expression using the specific inhibitor RO4929097 resulted in decreased expression levels of downstream effectors HES1 and HEY1. Interestingly, pretreatment with RO4929097 for 24 h caused Molm-13 cells to become resistant to AZA and attenuated the cytotoxic activity of NK and T cells. Levels of IFN-γ and TNF-α dramatically decreased in the supernatants of NK and T cell cultures treated with RO4929097 alone or in combination with AZA, compared to cells exposed to AZA alone [5] (Figure 4A).

Additionally, AZA inactivates the phosphatidylinositol 3-kinase (PI3K)/Akt pathway, which typically regulates cell survival over cell death programs and exhibits properties characteristic of malignant tumors. Several studies have reported that phosphorylation of Akt at Thr308 serves as a marker of an aggressive phenotype and poor prognosis, particularly in AML patients, as its activation contributes to chemotherapy resistance [44]. Interestingly, AZA’s inhibition of Akt activation at the Thr308 residue contributed to the initiation of apoptotic pathways and the activation of poly(ADP-ribose) polymerase (PARP) in AML cell lines. Furthermore, PARP cleavage was increased following combined treatment with AZA and Ara-C in AML cells [4] (Figure 4B).

Furthermore, AZA also exerts a pro-apoptotic effect on AML cells by targeting the c-jun-activation-domain binding protein-1 and thioredoxin axis (Jab1-Trx), a key cellular process in the pathogenesis of AML, particularly in those with the monocyte phenotype (AML-M5). Jab1 overexpression has been identified as a promoter of tumorigenesis by activating various substrates such as cyclin E, Smad 4/7, and p53, or by blocking p27 [4], in a variety of malignant diseases [45]. Additionally, Jab1 is overexpressed in AML and is associated with lower overall survival in patients. Mechanistically, Jab1 regulates Trx transcriptionally and stabilizes the Trx protein by interacting with it. Furthermore, reducing Jab1 levels inhibits leukemia cell growth in both in vitro and in vivo models [45] (Figure 4C). The expression of Jab1/Trx was reduced by AZA in a dose-dependent manner, and this effect was enhanced in AML cells exposed to combined AZA and Ara-C treatment. Further exploration using Jab1 siRNA in AML cells revealed that the decrease in phosphorylated Akt was induced by Jab1 reduction, with this effect being further intensified by additional treatment with either AZA or Ara-C [4](Figure 4D). The molecular basis underlying the antileukemic activity of AZA was further explored by LC-MS analysis. In Molm-13, THP-1, and primary AML patient cells (AML-PC) treated with AZA, differentially expressed proteins (DEPs) were identified, with roles in antioxidant activity and immune response. Specifically, upregulation of Prdx2 (Peroxiredoxin 2) and Prdx3 (Peroxiredoxin 3), key enzymes in the ROS-scavenging Prdx system, was observed. Protein-protein interaction network analysis using STRING revealed that Prdx3 interacts with several antioxidant enzymes, including catalase (CAT) and SOD2, which are typically upregulated after AZA treatment. Notably, AZA-induced expression of Prdx3, combined with the upregulation of SOD2 and CAT, synergistically decreases intracellular ROS levels in AML cells [5] (Figure 4E).

The cytotoxic activity of AZA in AML involves the coordinated modulation of key signaling pathways, including Notch and PI3K/Akt, and redox homeostasis. These mechanisms underlie its pro-apoptotic effects and enhance its synergy with conventional therapies, reinforcing its potential as a multi-targeted therapeutic agent in AML.

### 4.3. Enhancing AML Treatment: AZA as a Sensitizer to Cytarabine’s Antileukemic Activity

AML remains a challenging cancer to treat, despite the longstanding use of Cytarabine (Ara-C) as a cornerstone of therapy [46]. Recent research indicates that combining Ara-C with other agents may enhance its efficacy. Azacitidine is one such promising agent, showing potential to boost the antileukemic effects of Ara-C, particularly when used in combination at suboptimal doses [47]. AZA supports the antileukemic activity of Cytarabine (1-β-D-arabinofuranosylcytosine), a drug that has been a standard treatment for AML for over 40 years [48]. In vitro studies have demonstrated that AZA can sensitize AML cells to Ara-C (5 μmol/L) when applied at suboptimal doses of 1 mmol/L (<IC20) for 24 h. Under these conditions, the viability of AML cells (U937, THP-1, KG-1, NB4, HL-60) was significantly reduced compared to those exposed to Ara-C alone. These findings are promising and provide a strong basis for improving the effectiveness of Ara-C in AML treatment [4].

By sensitizing AML cells to cytarabine, AZA may enhance treatment efficacy even at subtherapeutic drug levels. This synergistic interaction supports its integration into combination regimens aimed at overcoming chemoresistance and improving therapeutic outcomes in AML.

### 4.4. Effective In Vivo Validation of AZA in Suppressing AML Tumorigenicity and Enhancing Immunologic Response Across Mouse Models

**Figure 5:**
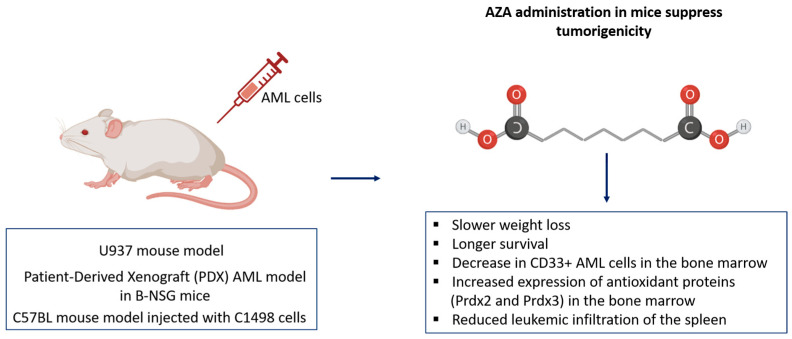
Effects of Azacitidine (AZA) in AML mouse models. AZA treatment reduced tumor growth, improved survival, and decreased CD33^+^ AML cells in the bone marrow. Enhanced oxidative stress markers and increased antioxidant proteins (Prdx2 and Prdx3) were observed, along with reduced leukemic infiltration of the spleen.

The antitumor effects of AZA on AML have been demonstrated across several mouse models. Initially, AZA’s ability to suppress tumorigenicity was shown in a U937 mouse model, where AZA treatment led to a significant reduction in tumor growth compared to controls, attributed to decreased expression of Jab1 and p-Akt in leukemic cells [4]. Further validation was achieved using a Patient-Derived Xenograft (PDX) AML model in B-NSG mice. In this model, mice injected with AML patient cells were treated with either AZA or saline. AZA-treated mice exhibited slower weight loss, longer survival, and a significant decrease in CD33^+^ AML cells in the bone marrow, indicating disease remission. AZA also improved oxidative stress markers and increased the expression of antioxidant proteins (Prdx2 and Prdx3) in the bone marrow, aligning with previous in vitro results [5]. Additionally, AZA’s effects were confirmed in a C57BL mouse model injected with C1498 cells. Mice treated with AZA (10 mg/kg) every three days for three weeks demonstrated delayed tumorigenicity and reduced leukemic infiltration of the spleen. Flow cytometric analysis revealed a significant increase in CD3-CD56^+^ NK cells and CD4^+^CD8^+^ T cells in the AZA-treated group, compared to controls. This enhanced cytotoxicity was associated with increased secretion of IFN-γ and TNF in the plasma, contributing to extended survival of AML mice [5] (Figure 5).

In vivo studies confirm AZA’s antileukemic effects, demonstrating reduced tumor burden, improved survival, and enhanced immune activation. These results validate the translational potential of AZA as both a cytotoxic and immunomodulatory agent in AML therapy.

## 5. Conclusions

AZA has emerged as a promising therapeutic candidate in the treatment of acute myeloid leukemia (AML), showing significant antiproliferative, pro-apoptotic, and immunomodulatory effects. Through the regulation of redox balance, modulation of Notch and PI3K/Akt signaling pathways, and enhancement of chemotherapy sensitivity, AZA has shown the potential to overcome key mechanisms of AML progression and chemoresistance. Notably, its ability to selectively target leukemia cells while sparing healthy hematopoietic cells makes it an attractive option for future therapeutic development.

Despite these promising findings, several challenges must be addressed before clinical translation. While preclinical studies have established AZA’s efficacy in vitro and in vivo, further research is needed to determine its pharmacokinetic properties, optimal dosing regimens, and potential toxicities in human subjects. Additionally, large-scale clinical trials are essential to validate AZA’s safety and therapeutic benefit in AML patients. Future investigations should also explore AZA’s combinatorial potential with existing chemotherapeutic agents, targeted therapies, and immunotherapies to enhance treatment efficacy and minimize resistance.

In conclusion, AZA represents an exciting and innovative approach to AML treatment. By leveraging its multiple mechanisms of action, it has the potential to improve therapeutic outcomes and reduce chemoresistance in AML patients. Continued research efforts will be critical in advancing AZA from preclinical promise to a viable clinical option, ultimately contributing to the evolution of AML treatment strategies.

## Figures and Tables

**Table 1:** Antileukemic Effects of AZA on Various AML Cell Lines and Primary Cells. The table provides an overview of the antileukemic effects of AZA across various AML cell lines and primary cells, detailing the concentration and duration of AZA treatment. Key findings include reductions in cell viability, colony formation, and the induction of apoptosis. Additionally, the table highlights the impact of AZA-treated immune cell supernatants on AML cells, emphasizing the enhanced cytolytic response. Relevant references are cited for each study.

Cell Type	Hours of AZA Treatment	AZA Concentration	Biological Effects	Ref.
U937 THP-1 KG-1 NB-4 HL-60	72	IC50: 1.4 mM 1.2 mM 1.7 mM 1.7 mM 1.9 mM	Reduction in cell viability, Induction of apoptosis	[4]
U937 THP-1 KG-1 NB-4 HL-60	72	3.0 mM	Reduction in colony formation	[4]
U937 HL-60 Molm-13 human AML cells	48	5.0 mM	Reduction in cell viability, Induction of apoptosis	[6]
THP-1 U937 human AML cells	6	Co-culture with 10 µM AZA-treated NK and T cell supernatants	Reduction in cell viability, Enhanced expression of cytolysis-related receptors	[6]
Healthy PBMCs	72	5–10 mM	No cytolytic effects observed	[4]

## Data Availability

No new data were created.

## Abbreviations

Abbreviation	Full Term
AML	Acute Myeloid Leukemia
AZA	Azelaic Acid
ROS	Reactive Oxygen Species
PBMC	Peripheral Blood Mononuclear Cell
HSC	Hematopoietic Stem Cell
LSC	Leukemic Stem Cell
CMP	Common Myeloid Progenitor
CLP	Common Lymphoid Progenitor
GMP	Granulocyte-Macrophage Progenitor
MEP	Megakaryocyte-Erythroid Progenitor
PDX	Patient-Derived Xenograft
NF-κB	Nuclear Factor kappa-light-chain-enhancer of activated B cells
PI3K	Phosphatidylinositol 3-Kinase
Akt	Protein Kinase B
PARP	Poly(ADP-ribose) Polymerase
Trx	Thioredoxin
TrxR	Thioredoxin Reductase
TXNIP	Thioredoxin-Interacting Protein
RNR	Ribonucleotide Reductase
dNTP	Deoxyribonucleotide Triphosphate
SOD	Superoxide Dismutase
GSH	Glutathione
Prdx	Peroxiredoxin
CAT	Catalase
MMP	Mitochondrial Membrane Potential
NK	Natural Killer (cells)
TGF-β	Transforming Growth Factor Beta
IFN-γ	Interferon Gamma
TNF-α	Tumor Necrosis Factor Alpha
ELN	European LeukemiaNet
WHO	World Health Organization
ICC	International Consensus Classification
MRD	Minimal Residual Disease
FLT3–ITD	FMS-like Tyrosine Kinase 3–Internal Tandem Duplication
IDH	Isocitrate Dehydrogenase
BCL-2	B-cell Lymphoma 2
HMA	Hypomethylating Agent
DAs	Dicarboxylic Acids
PPARγ	Peroxisome Proliferator-Activated Receptor Gamma
PPP	Pentose Phosphate Pathway
TF	Transcription Factor
